# skater: an R package for SNP-based kinship analysis, testing, and evaluation

**DOI:** 10.12688/f1000research.76004.1

**Published:** 2022-01-07

**Authors:** Stephen D. Turner, V.P. Nagraj, Matthew Scholz, Shakeel Jessa, Carlos Acevedo, Jianye Ge, August E. Woerner, Bruce Budowle

**Affiliations:** 1 Signature Science, LLC., Austin, TX, 78759, USA; 2 Center for Human Identification, Department of Microbiology, Immunology, and Genetics, University of North Texas Health Science Center, Fort Worth, TX, 76107, USA

**Keywords:** bioinformatics, kinship, R, genealogy, SNPs, single nucleotide polymorphisms, relatedness

## Abstract

**Motivation**: SNP-based kinship analysis with genome-wide relationship estimation and IBD segment analysis methods produces results that often require further downstream process- ing and manipulation. A dedicated software package that consistently and intuitively imple- ments this analysis functionality is needed.

**Results**: Here we present the skater R package for SNP-based kinship analysis, testing, and evaluation with R. The skater package contains a suite of well-documented tools for importing, parsing, and analyzing pedigree data, performing relationship degree inference, benchmarking relationship degree classification, and summarizing IBD segment data.

**Availability**: The skater package is implemented as an R package and is released under the MIT license at https://github.com/signaturescience/skater. Documentation is available at https://signaturescience.github.io/skater.

**R version**: R version 4.0.4 (2021-02-15)

**skater package version**: 0.1.0

## Introduction

Inferring familial relationships between individuals using genetic data is a common practice in population genetics, medical genetics, and forensics. There are multiple approaches to estimating relatedness between samples, including genome-wide measures, such as those implemented in Plink ^1^ or KING, ^2^ and methods that rely on identity by descent (IBD) segment detection, such as GERMLINE, ^3^ hap-IBD, ^4^ and IBIS. ^5^ Recent efforts focusing on benchmarking these methods ^6^ have been aided by tools for simulating pedigrees and genome-wide SNP data. ^7^ Analyzing results from genome-wide SNP-based kinship analysis or comparing analyses to simulated data for benchmarking have to this point required writing one-off analysis functions or utility scripts that are seldom distributed with robust documentation, test suites, or narrative examples of usage. There is a need in the field for a well-documented software package with a consistent design and API that contains functions to assist with downstream manipulation, benchmarking, and analysis of SNP-based kinship assessment methods. Here we present the skater package for **S**NP-based **k**inship **a**nalysis, **t**esting, and **e**valuation with **R**.

## Methods

### Implementation

The skater package provides an intuitive collection of analysis and utility functions for SNP-based kinship analysis. Functions in the package include tools for importing, parsing, and analyzing pedigree data, performing relationship degree inference, benchmarking relationship degree classification, and summarizing IBD segment data, described in full in the *Use Cases* section below. The package adheres to “tidy” data analysis principles, and builds upon the tools released under the tidyverse R ecosystem. ^8^

The skater package is hosted in the Comprehensive R Archive Network (CRAN) which is the main repository for R packages: http://CRAN.R-project.org/package=skater. Users can install skater in R by executing the following code:

install.packages("skater")



Alternatively, the development version of skater is available on GitHub at https://github.com/signaturescience/skater. The development version may contain new features which are not yet available in the version hosted on CRAN. This version can be installed using the install_github() function in the devtools package:

install.packages("devtools")
devtools::install_github("signaturescience/skater", build_vignettes=TRUE)



When installing skater, other packages which skater depends on are automatically installed, including magritr, tibble, dplyr, tidyr, readr, purrr, kinship2, corrr, rlang, and others.

### Operation

Minimal system requirements for installing and using skater include R (version 3.0.0 or higher) and several tidyverse packages ^8^ that many R users will already have installed. Use cases are demonstrated in detail below. In summary, the skater package has functions for:

• Reading in various output files produced by commonly used tools in SNP-based kinship analysis
• Pedigree parsing, manpulation, and analysis
• Relationship degree inference
• Benchmarking and assessing relationship classification accuracy
• IBD segment analysis post-processing

A comprehensive reference for all the functions in the skater package is available at https://signaturescience.github.io/skater/.

## Use cases

The skater package provides a collection of analysis and utility functions for **S**NP-based **k**inship **a**nalysis, **t**esting, and **e**valuation as an **R** package. Functions in the package include tools for working with pedigree data, performing relationship degree inference, assessing classification accuracy, and summarizing IBD segment data.

library(skater)



### Pedigree parsing, manipulation, and analysis

Pedigrees define familial relationships in a hierarchical structure. One of the common formats used by PLINK ^1^ and other genetic analysis tools is the .fam file. A .fam file is a tabular format with one row per individual and columns for unique IDs of the mother, father, and the family unit. The package includes read_fam() to read files in this format:

famfile <- system.file("extdata", "3gens.fam", package="skater", mustWork=TRUE)
fam <- read_fam(famfile)
fam


## # A tibble: 64 x 6
##   fid    id          dadid        momid       sex affected
##   <chr>   <chr>        <chr>        <chr>      <int>   <int>
## 1  testped1 testped1_g1-b1-s1 0          0           1     1
## 2  testped1 testped1_g1-b1-i1 0          0           2     1
## 3  testped1 testped1_g2-b1-s1 0          0           1     1
## 4  testped1 testped1_g2-b1-i1 testped1_g1-b1-s1 testped1_g1-b1-i1 2     1
## 5  testped1 testped1_g2-b2-s1 0          0           1     1
## 6  testped1 testped1_g2-b2-i1 testped1_g1-b1-s1 testped1_g1-b1-i1  2     1
## 7  testped1 testped1_g3-b1-i1 testped1_g2-b1-s1 testped1_g2-b1-i1  2     1
## 8  testped1 testped1_g3-b2-i1 testped1_g2-b2-s1 testped1_g2-b2-i1  1     1
## 9  testped2 testped2_g1-b1-s1 0          0           2     1
## 10 testped2 testped2_g1-b1-i1 0          0           1     1
## #… with 54 more rows



Family structures imported from .fam formated files can then be translated to the pedigree structure used by the kinship2 package. ^9^ The “fam” format may include multiple families, and the fam2ped() function will collapse them all into a tibble with one row per family:

peds <- fam2ped(fam)
peds


## # A tibble: 8 x 3
##   fid    data          ped
##   <chr>   <list>        <list>
## 1 testped1 <tibble [8 x 5]> <pedigree>
## 2 testped2 <tibble [8 x 5]> <pedigree>
## 3 testped3 <tibble [8 x 5]> <pedigree>
## 4 testped4 <tibble [8 x 5]> <pedigree>
## 5 testped5 <tibble [8 x 5]> <pedigree>
## 6 testped6 <tibble [8 x 5]> <pedigree>
## 7 testped7 <tibble [8 x 5]> <pedigree>
## 8 testped8 <tibble [8 x 5]> <pedigree>



In the example above, the resulting tibble is nested by family ID. The data column contains the individual family information, while the ped column contains the pedigree object for that family. Using standard tidyverse operations, the resulting tibble can be unnested for any particular family:

peds %>%
 dplyr::filter(fid=="testped1") %>%
 tidyr::unnest(cols=data)


## # A tibble: 8 x 7
##   fid    id           dadid        momid   sex affected   ped
##   <chr>   <chr>        <chr>        <chr>  <int>   <dbl> <list>
## 1 testped1 testped1_g1-b1-s1 <NA>         <NA>      1       1 <pedig~
## 2 testped1 testped1_g1-b1-i1 <NA>         <NA>      2       1 <pedig~
## 3 testped1 testped1_g2-b1-s1 <NA>         <NA>      1       1 <pedig~
## 4 testped1 testped1_g2-b1-i1 testped1_g1-b1-s1 testped1_~  2       1 <pedig~
## 5 testped1 testped1_g2-b2-s1 <NA>         <NA>      1       1 <pedig~
## 6 testped1 testped1_g2-b2-i1 testped1_g1-b1-s1 testped1_~  2       1 <pedig~
## 7 testped1 testped1_g3-b1-i1 testped1_g2-b1-s1 testped1_~  2       1 <pedig~
## 8 testped1 testped1_g3-b2-i1 testped1_g2-b2-s1 testped1_~  1       1 <pedig~



A single pedigree can also be inspected or visualized (standard base R plot arguments such as mar or cex can be used to adjust aesthetics):

peds$ped[[1]]


## Pedigree object with 8 subjects
## Bit size= 4


plot(peds$ped[[1]], mar=c(1,4,1,4), cex=.7)



**Figure 1.**
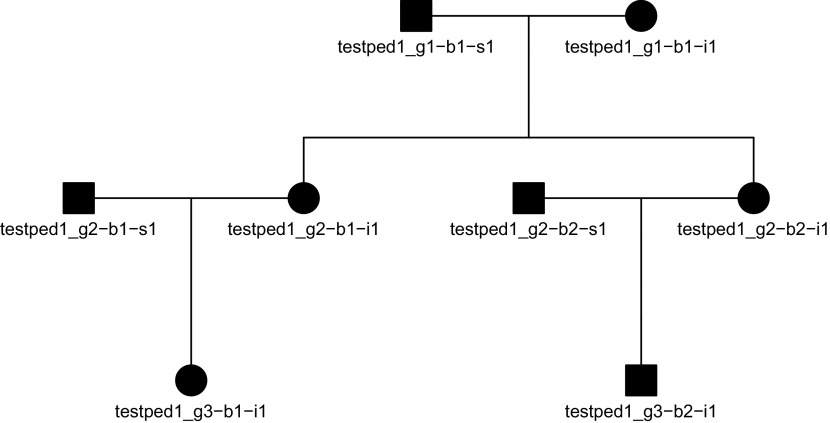
Pedigree diagram for the first family in the pedigree shown in the code above.

The plot_pedigree() function from skater will iterate over a list of pedigree objects, writing a multi-page PDF, with each page containing a pedigree from family:

plot_pedigree(peds$ped, file="3gens.ped.pdf")



The ped2kinpair() function takes a pedigree object and produces a pairwise list of relationships between all individuals in the data with the expected kinship coefficients for each pair.

The function can be run on a single family:

ped2kinpair(peds$ped[[1]])


## # A tibble: 36 x 3
##   id1          id2            k
##   <chr>         <chr>        <dbl>
##  1 testped1_g1-b1-s1 testped1_g1-b1-s1 0.5
##  2 testped1_g1-b1-i1 testped1_g1-b1-s1 0
##  3 testped1_g1-b1-s1 testped1_g2-b1-s1 0
##  4 testped1_g1-b1-s1 testped1_g2-b1-i1 0.25
##  5 testped1_g1-b1-s1 testped1_g2-b2-s1 0
##  6 testped1_g1-b1-s1 testped1_g2-b2-i1 0.25
##  7 testped1_g1-b1-s1 testped1_g3-b1-i1 0.125
##  8 testped1_g1-b1-s1 testped1_g3-b2-i1 0.125
##  9 testped1_g1-b1-i1 testped1_g1-b1-i1 0.5
## 10 testped1_g1-b1-i1 testped1_g2-b1-s1 0
## # … with 26 more rows



This function can also be mapped over all families in the pedigree:

kinpairs <-
 peds %>%
 dplyr::mutate(pairs=purrr::map(ped, ped2kinpair)) %>%
 dplyr::select(fid, pairs) %>%
 tidyr::unnest(cols=pairs)
kinpairs


## # A tibble: 288 x 4
##   fid     id1         id2            k
##   <chr>    <chr>        <chr>        <dbl>
##  1 testped1 testped1_g1-b1-s1 testped1_g1-b1-s1 0.5
##  2 testped1 testped1_g1-b1-i1 testped1_g1-b1-s1 0
##  3 testped1 testped1_g1-b1-s1 testped1_g2-b1-s1 0
##  4 testped1 testped1_g1-b1-s1 testped1_g2-b1-i1 0.25
##  5 testped1 testped1_g1-b1-s1 testped1_g2-b2-s1 0
##  6 testped1 testped1_g1-b1-s1 testped1_g2-b2-i1 0.25
##  7 testped1 testped1_g1-b1-s1 testped1_g3-b1-i1 0.125
##  8 testped1 testped1_g1-b1-s1 testped1_g3-b2-i1 0.125
##  9 testped1 testped1_g1-b1-i1 testped1_g1-b1-i1 0.5
## 10 testped1 testped1_g1-b1-i1 testped1_g2-b1-s1 0
## # … with 278 more rows



Note that this maps ped2kinpair() over all ped objects in the input tibble, and that relationships are not shown for between-family relationships.

### Relationship degree inference and benchmarking

The skater package includes functions to translate kinship coefficients to relationship degrees. The kinship coefficients could come from ped2kinpair() or other kinship estimation software.

The dibble() function creates a **d**egree **i**nference tibble, with degrees up to the specified max_degree (default=3), expected kinship coefficient, and lower (l) and upper (u) inference ranges as defined in Manichaikul et al. ^2^ Degree 0 corresponds to self/identity/monozygotic twins, with an expected kinship coefficient of 0.5, with inference range >=0.354. Anything beyond the maximum degree resolution is considered unrelated (degree NA). Note also that while the theoretical upper boundary for the kinship coefficient is 0.5, the inference range for 0-degree (same person or identical twins) extends to 1 to allow for floating point arithmetic and stochastic effects resulting in kinship coefficients above 0.5.

dibble()


## # A tibble: 5 x 4
##  degree   k     l     u
##  <int>  <dbl>   <dbl>  <dbl>
## 1   0 0.5    0.354  1
## 2   1 0.25   0.177  0.354
## 3   2 0.125   0.0884  0.177
## 4   3 0.0625  0.0442 0.0884
## 5  NA 0     -1     0.0442



The degree inference max_degree default is 3. Change this argument to allow more granular degree inference ranges:

dibble (max_degree = 5)


## # A tibble: 7 x 4
##  degree   k    l    u
##  <int>  <dbl>  <dbl>  <dbl>
## 1   0 0.5   0.354  1
## 2   1 0.25  0.177  0.354
## 3   2 0.125  0.0884 0.177
## 4   3 0.0625 0.0442 0.0884
## 5   4 0.0312 0.0221 0.0442
## 6   5 0.0156 0.0110 0.0221
## 7  NA 0    -1     0.0110



Note that the distance between relationship degrees becomes smaller as the relationship degree becomes more distant. The dibble() function will emit a warning with max_degree >=10, and will stop with an error at >=12.

The kin2degree() function infers the relationship degree given a kinship coefficient and a max_degree up to which anything more distant is treated as unrelated. Example first degree relative:

kin2degree(.25, max_degree=3)
## [1] 1



Example 4th degree relative, but using the default max_degree resolution of 3:

kin2degree(.0312, max_degree=3)
## [1] NA



Example 4th degree relative, but increasing the degree resolution:

kin2degree(.0312, max_degree=5)
## [1] 4



The kin2degree() function is vectorized over values of k, so it can be used inside of a mutate on a tibble of kinship coefficients:

# Get two pairs from each type of relationship we have in kinpairs:
kinpairs_subset <-
 kinpairs %>%
 dplyr::group_by(k) %>%
 dplyr::slice(1:2)
kinpairs_subset


## # A tibble: 10 x 4
## # Groups:  k [5]
##   fid     id1          id2             k
##   <chr>    <chr>        <chr>          <dbl>
##  1 testped1 testped1_g1-b1-i1 testped1_g1-b1-s1 0
##  2 testped1 testped1_g1-b1-s1 testped1_g2-b1-s1 0
##  3 testped1 testped1_g3-b1-i1 testped1_g3-b2-i1 0.0625
##  4 testped2 testped2_g3-b1-i1 testped2_g3-b2-i1 0.0625
##  5 testped1 testped1_g1-b1-s1 testped1_g3-b1-i1 0.125
##  6 testped1 testped1_g1-b1-s1 testped1_g3-b2-i1 0.125
##  7 testped1 testped1_g1-b1-s1 testped1_g2-b1-i1 0.25
##  8 testped1 testped1_g1-b1-s1 testped1_g2-b2-i1 0.25
##  9 testped1 testped1_g1-b1-s1 testped1_g1-b1-s1 0.5
## 10 testped1 testped1_g1-b1-i1 testped1_g1-b1-i1 0.5


# Infer degree out to third degree relatives:
kinpairs_subset %>%
 dplyr::mutate (degree=kin2degree(k, max_degree=3))


## # A tibble: 10 x 5
## # Groups:  k [5]
##   fid     id1          id2            k  degree
##   <chr>    <chr>        <chr>         <dbl>  <int>
##  1 testped1 testped1_g1-b1-i1 testped1_g1-b1-s1 0      NA
##  2 testped1 testped1_g1-b1-s1 testped1_g2-b1-s1 0      NA
##  3 testped1 testped1_g3-b1-i1 testped1_g3-b2-i1 0.0625    3
##  4 testped2 testped2_g3-b1-i1 testped2_g3-b2-i1 0.0625    3
##  5 testped1 testped1_g1-b1-s1 testped1_g3-b1-i1 0.125    2
##  6 testped1 testped1_g1-b1-s1 testped1_g3-b2-i1 0.125    2
##  7 testped1 testped1_g1-b1-s1 testped1_g2-b1-i1 0.25     1
##  8 testped1 testped1_g1-b1-s1 testped1_g2-b2-i1 0.25     1
##  9 testped1 testped1_g1-b1-s1 testped1_g1-b1-s1 0.5     0
## 10 testped1 testped1_g1-b1-i1 testped1_g1-b1-i1 0.5     0



### Benchmarking degree classification

Once estimated kinship is converted to degree, it may be of interest to compare the inferred degree to truth. When aggregated over many relationships and inferences, this approach can help benchmark performance of a particular kinship analysis method.

The skater package adapts a confusion_matrix() function from Clark ^10^ to provide standard contingency table metrics (e.g. sensitivity, specificity, PPV, precision, recall, F1, etc.) with a new reciprocal RMSE (R-RMSE) metric. The confusion_matrix() function on its own outputs a list with four objects:

1. A tibble with calculated accuracy, lower and upper bounds, the guessing rate and p-value of the accuracy vs. the guessing rate.
2. A tibble with contingency table statistics calculated for each class. Details on the statistics calculated for each class can be reviewed on the help page for ?confusion_matrix.
3. A matrix with the contingency table object itself.
4. A vector with the reciprocal RMSE (R-RMSE). The R-RMSE represents an alternative to classification accuracy when benchmarking relationship degree estimation and is calculated using the formula in (1). Taking the reciprocal of the target and predicted degree results in larger penalties for more egregious misclassifications (e.g., classifying a first-degree relative pair as second degree) than misclassifications at more distant relationships (e.g., misclassifying a fourth-degree relative pair as fifth-degree). The +0.5 adjustment prevents division-by-zero when a 0th-degree (identical) relative pair is introduced.

$$\sqrt{\frac{\sum_{i=1}^{k}{\left(\frac{1}{\text{Target}+0.5}-\frac{1}{\text{Predicted}+0.5}\right)}^{2}}{k}}$$ (1)

To illustrate the usage, this example will start with the kinpairs data from above and randomly flip ~20% of the true relationship degrees:

# Function to randomly flip levels of a factor (at 20%, by default)
randomflip <- function(x, p=.2) ifelse(runif(length(x))<p, sample(unique(x)), x)


# Infer degree (truth/target) using kin2degree, then randomly flip 20% of them
set.seed(42)
kinpairs_inferred <- kinpairs %>%
 dplyr::mutate(degree_truth=kin2degree(k, max_degree=3)) %>%
 dplyr::mutate(degree_truth=tidyr::replace_na(degree_truth, "unrelated")) %>%
 dplyr::mutate(degree_inferred=randomflip (degree_truth))
kinpairs_inferred


## # A tibble: 288 x 6
##   fid    id1          id2         k    degree_truth degree_inferred
##   <chr>   <chr>        <chr>        <dbl> <chr>     <chr>
## 1 testped1 testped1_g1-b1-s1 testped1_g1-b1-s1 0.5  0       0
## 2 testped1 testped1_g1-b1-i1 testped1_g1-b1-s1 0   unrelated  unrelated
## 3 testped1 testped1_g1-b1-s1 testped1_g2-b1-s1 0   unrelated  unrelated
## 4 testped1 testped1_g1-b1-s1 testped1_g2-b1-i1 0.25 1       1
## 5 testped1 testped1_g1-b1-s1 testped1_g2-b2-s1 0   unrelated  unrelated
## 6 testped1 testped1_g1-b1-s1 testped1_g2-b2-i1 0.25 1       1
## 7 testped1 testped1_g1-b1-s1 testped1_g3-b1-i1 0.125 2       2
## 8 testped1 testped1_g1-b1-s1 testped1_g3-b2-i1 0.125 2       1
## 9 testped1 testped1_g1-b1-i1 testped1_g1-b1-i1 0.5  0       0
## 10 testped1 testped1_g1-b1-i1 testped1_g2-b1-s1 0   unrelated  unrelated
## #… with 278 more rows



Next, running the confusion_matrix() function will return all four objects noted above:

confusion_matrix(prediction = kinpairs_inferred$degree_inferred,
         target = kinpairs_inferred$degree_truth)


## $Accuracy
## # A tibble: 1 x 5
##  Accuracy ‘Accuracy LL‘ ‘Accuracy UL‘ ‘Accuracy Guessing‘ ‘Accuracy P-value‘
##    <dbl>      <dbl>      <dbl>          <dbl>          <dbl>
## 1   0.812      0.763      0.856          0.333        1.09e-62
##
## $Other
## # A tibble: 6 x 15
##   Class      N ‘Sensitivity/Re~ ‘Specificity/TN~ ‘PPV/Precision‘ NPV  ‘F1/Dice‘
##   <chr>  <dbl>        <dbl>        <dbl>      <dbl> <dbl>   <dbl>
## 1 0     64         0.75        0.964      0.857 0.931   0.8
## 2 1     72         0.806        0.944      0.829 0.936   0.817
## 3 2     48         0.833        0.967      0.833 0.967   0.833
## 4 3      8         0.75        0.936       0.25  0.992   0.375
## 5 unrelated 96         0.854        0.958      0.911 0.929   0.882
## 6 Average  57.6        0.799        0.954      0.736 0.951   0.741
## # … with 8 more variables: Prevalence <dbl>, Detection Rate <dbl>,
## #  Detection Prevalence <dbl>, Balanced Accuracy <dbl>, FDR <dbl>, FOR <dbl>,
## #  FPR/Fallout <dbl>, FNR <dbl>
##
## $Table
##       Target
## Predicted  0  1  2 3 unrelated
##  0      48  4  2 1      1
##  1      5 58  4 0      3
##  2      0  3 40 1      4
##  3      8  4  0 6      6
##  unrelated 3  3  2 0     82
##
## $recip_rmse
## [1] 0.4665971



Standard tidyverse functions such as purrr::pluck() can be used to isolate just the contingency table:

confusion_matrix(prediction = kinpairs_inferred$degree_inferred,
           target = kinpairs_inferred$degree_truth) %>%
 purrr::pluck("Table")


##       Target
## Predicted  0  1  2 3 unrelated
##  0      48  4  2 1      1
##  1      5 58  4 0      3
##  2      0  3 40 1       4
##  3      8  4  0 6       6
##  unrelated 3  3  2 0      82



The confusion_matrix() function includes an argument to output in a tidy ( longer=TRUE) format, and the example below illustrates how to spread contingency table statistics by class:

confusion_matrix(prediction = kinpairs_inferred$degree_inferred,
          target = kinpairs_inferred$degree_truth,
          longer = TRUE) %>%
 purrr::pluck("Other") %>%
 tidyr::spread (Class, Value) %>%
 dplyr::relocate (Average, .after=dplyr::last_col()) %>%
 dplyr::mutate_if(rlang::is_double, signif, 2)


## # A tibble: 14 x 7
##   Statistic           ‘0‘   ‘1‘   ‘2‘   ‘3‘  unrelated  Average
##   <chr>             <dbl>  <dbl>  <dbl>  <dbl>    <dbl>   <dbl>
##  1 Balanced Accuracy      0.86  0.88  0.9   0.84     0.91   0.88
##  2 Detection Prevalence    0.19  0.24  0.17  0.083    0.31   0.2
##  3 Detection Rate       0.17  0.2   0.14  0.021    0.28   0.16
##  4 F1/Dice            0.8   0.82  0.83  0.38     0.88   0.74
##  5 FDR              0.14  0.17  0.17  0.75     0.089   0.26
##  6 FNR              0.25  0.19  0.17  0.25     0.15   0.2
##  7 FOR              0.069  0.064  0.033  0.0076    0.071   0.049
##  8 FPR/Fallout         0.036  0.056  0.033  0.064     0.042   0.046
##  9 N              64   72   48    8      96    58
## 10 NPV              0.93  0.94  0.97  0.99     0.93   0.95
## 11 PPV/Precision        0.86  0.83  0.83  0.25     0.91   0.74
## 12 Prevalence          0.22  0.25  0.17  0.028    0.33   0.2
## 13 Sensitivity/Recall/TPR  0.75  0.81  0.83  0.75     0.85   0.8
## 14 Specificity/TNR       0.96  0.94  0.97  0.94     0.96   0.95



### IBD segment analysis

Tools such as hap-IBD, ^4^ and IBIS ^5^ detect shared IBD segments between individuals. The skater package includes functionality to take those IBD segments, compute shared genomic centimorgan (cM) length, and converts that shared cM to a kinship coefficient. In addition to inferred segments, these functions can estimate “truth” kinship from simulated IBD segments. ^7^ The read_ibd() function reads pairwise IBD segments from IBD inference tools and from simulated IBD segments. The read_map() function reads in genetic map in a standard format which is required to translate the total centimorgans shared IBD to a kinship coefficient using the ibd2kin() function. See ?read_ibd and ?read_map for additional details on expected format.

The read_ibd() function reads in the pairwise IBD segment format. Input to this function can either be inferred IBD segments from hap-IBD ( source="hapibd") or simulated segments ( source="pedsim"). The first example below uses data in the hap-ibd output format:

hapibd_filepath <- system.file("extdata", "GBR.sim.ibd.gz",
                   package="skater")
hapibd_seg <- read_ibd(hapibd_filepath, source = "hapibd")
hapibd_seg


## # A tibble: 3,954 x 6
##   id1          id2           chr   start    end  length
##   <chr>         <chr>        <dbl>   <dbl>   <dbl>  <dbl>
##  1 testped1_g1-b1-s1 testped1_g3-b1-i1   1 197661576 234863602  47.1
##  2 testped1_g2-b2-i1 testped1_g3-b1-i1   1 197661576 231017545  39.8
##  3 testped1_g3-b1-i1 testped1_g3-b2-i1   1 197661576 212799139  20.3
##  4 testped3_g1-b1-s1 testped3_g3-b2-i1   1  2352146  10862397  17.7
##  5 testped3_g2-b2-i1 testped3_g3-b2-i1   1  2352146  10862397  17.7
##  6 testped1_g1-b1-s1 testped1_g2-b1-i1   1  3328659  64123868  86.4
##  7 testped1_g1-b1-s1 testped1_g3-b1-i1   1  3328659  33476811  51.2
##  8 testped1_g2-b2-s1 testped1_g3-b2-i1   1  5003504  32315147  45.9
##  9 testped2_g1-b1-i1 testped2_g3-b1-i1   1 240810528 248578622  15.9
## 10 testped2_g1-b1-i1 testped2_g2-b2-i1   1 241186056 249170711  15.5
## # … with 3,944 more rows



In order to translate the shared genomic cM length to a kinship coefficient, a genetic map must first be read in with read_map(). Software for IBD segment inference and simulation requires a genetic map. The map loaded for kinship estimation should be the same one used for creating the shared IBD segment output. The example below uses a minimal genetic map that ships with skater:

gmap_filepath <- system.file("extdata", "sexspec-avg-min.plink.map",
                  package="skater")
gmap <- read_map(gmap_filepath)
gmap


## # A tibble: 28,726 x 3
##    chr value      bp
##   <dbl> <dbl>    <dbl>
##  1   1 0     752721
##  2   1 0.0292 1066029
##  3   1 0.0829 1099342
##  4   1 0.157  1106473
##  5   1 0.246  1152631
##  6   1 0.294  1314015
##  7   1 0.469  1510801
##  8   1 0.991  1612540
##  9   1 1.12  1892325
## 10   1 1.41  1916587
## # … with 28,716 more rows



The ibd2kin() function takes the segments and map file and outputs a tibble with one row per pair of individuals and columns for individual 1 ID, individual 2 ID, and the kinship coefficient for the pair:

ibd_dat <- ibd2kin(.ibd_data=hapibd_seg, .map=gmap)
ibd_dat


## # A tibble: 196 x 3
##   id1          id2           kinship
##   <chr>         <chr>          <dbl>
##  1 testped1_g1-b1-i1 testped1_g1-b1-s1 0.000316
##  2 testped1_g1-b1-i1 testped1_g2-b1-i1 0.261
##  3 testped1_g1-b1-i1 testped1_g2-b2-i1 0.263
##  4 testped1_g1-b1-i1 testped1_g2-b2-s1 0.000150
##  5 testped1_g1-b1-i1 testped1_g3-b1-i1 0.145
##  6 testped1_g1-b1-i1 testped1_g3-b2-i1 0.133
##  7 testped1_g1-b1-i1 testped2_g1-b1-i1 0.000165
##  8 testped1_g1-b1-i1 testped2_g1-b1-s1 0.000323
##  9 testped1_g1-b1-i1 testped2_g2-b1-i1 0.000499
## 10 testped1_g1-b1-i1 testped2_g2-b1-s1 0.000318
## # … with 186 more rows



## Summary

The skater R package provides a robust software package for data import, manipulation, and analysis tasks typically encountered when working with SNP-based kinship analysis tools. All package functions are internally documented with examples, and the package contains a vignette demonstrating usage, inputs, outputs, and interpretation of all key functions. The package contains internal tests that are automatically run with continuous integration via GitHub Actions whenever the package code is updated. The skater package is permissively licensed (MIT) and is easily extensible to accommodate outputs from new genome-wide relatedness and IBD segment methods as they become available.

### Software availability

1. Software available from: http://CRAN.R-project.org/package=skater .
2. Source code available from: https://github.com/signaturescience/skater .
3. Archived source code at time of publication: https://doi.org/10.5281/zenodo.5761996. ^11^
4. Software license: MIT License.

### Author information

SDT, VPN, and MBS developed the R package.

All authors contributed to method development.

SDT wrote the first draft of the manuscript.

All authors assisted with manuscript revision.

All authors read and approved the final manuscript.

## Competing interests

No competing interests were disclosed.

## Grant information

This work was supported in part by award 2019-DU-BX-0046 (Dense DNA Data for Enhanced Missing Persons Identification) to B.B., awarded by the National Institute of Justice, Office of Justice Programs, U.S. Department of Justice and by internal funds from the Center for Human Identification. The opinions, findings, and conclusions or recommendations expressed are those of the authors and do not necessarily reflect those of the U.S. Department of Justice.
