# Supplementary material for: skater: an R package for SNP-based kinship analysis, testing, and evaluation
Source: F1000Res. 2022 Jan 7;11:18. [Version 1] doi: 10.12688/f1000research.76004.1 (PMC8844523; doi:10.12688/f1000research.76004.1)
Supplement: Peer review history

# Peer review: Reviewer response for version 1

**DOI:** 10.5256/f1000research.79953.r119025

**Published:** 2022-02-14

**Authors:** Amy L. Williams

**Affiliations:** 1 Department of Computational Biology, Cornell University, Ithaca, NY, USA

This article describes an R package that provides a suite of tools for performing kinship and IBD segment analysis. Key features include methods for reading and analyzing pedigree data, inferring relationships, benchmarking relationship degree classification, and IBD segment analyses. The use cases in this article and the R reference manual on CRAN together provide exemplary documentation for skater. Users unfamiliar with pedigree-style analyses and/or who desire a clean, well documented interface for performing these analyses can find a great deal of utility in this R package.

Are the conclusions about the tool and its performance adequately supported by the findings presented in the article?

Yes

Is the rationale for developing the new software tool clearly explained?

Yes

Is the description of the software tool technically sound?

Yes

Are sufficient details of the code, methods and analysis (if applicable) provided to allow replication of the software development and its use by others?

Yes

Is sufficient information provided to allow interpretation of the expected output datasets and any results generated using the tool?

Yes

Reviewer Expertise:

Relatedness in large datasets.

I confirm that I have read this submission and believe that I have an appropriate level of expertise to confirm that it is of an acceptable scientific standard.

# Peer review: Reviewer response for version 1

**DOI:** 10.5256/f1000research.79953.r119020

**Published:** 2022-01-26

**Authors:** Magnus Dehli Vigeland

**Affiliations:** 1 Department of Medical Genetics, University of Oslo, Oslo, Norway

For many researchers, myself included, R is the language-of-choice when it comes to downstream analysis and tidying-up the output of other programs. This inevitably leads to a disorganized body of *ad hoc* scripts for parsing, cleaning, and manipulating various result files. Arguably, the best solution to this problem is to collect related scripts into an R package, which can be properly documented, version-controlled, and potentially shared with others. The **skater** package presented in this paper is precisely such a package, aimed at the users of several popular software for relatedness estimation, including KING, PLINK, hap-IBD, and others. A specific goal of the package is to provide tools that work consistently across these programs.

 The paper is written in clear language, well structured, and easy to follow. Parts of the paper resemble a user manual, including detailed code examples. I enjoyed playing around with the package, which is well organized and documented. In particular, I appreciate the thorough input checks and helpful error messages when things go wrong. The package may not offer a ton of novel methods yet, but I can certainly see myself using it the next time I run some of these relatedness programs.

 Below are a few issues I have with the manuscript.

1. It would be helpful to clarify what sort of applications skater is intended for. The introduction mentions "relationship inference in population genetics, medical genetics, and forensics", but that seems overly broad. The focus is on simple measures like the kinship coefficient and relatedness degree (rather than detailed coefficients and actual genealogical relationships) suggesting large-scale applications rather than small-scale pedigree analysis as e.g. in forensic case work. Indeed, all the programs referenced in the paper are intended for large-scale population studies. Furthermore, it seems to be an underlying assumption that all individuals are noninbred (suggested e.g. by the statement "the theoretical upper boundary for the kinship coefficient is 0.5"), and also that they are human (by the hardcoded 3560 cM genome length).
  To be clear, I think these limitations and assumptions are perfectly fine, but it would be better to state (or discuss) them more clearly.
2. A connection to "SNP-based methods" is mentioned repeatedly, but never really explained. Is there one? I note at least one of the cited programs (hap-IBD) is designed to work with sequencing data. Does it matter for skater how the kinship estimates were obtained?
3. The function `confusion_matrix()` produces an impressive array of summary stats when comparing inferred kinship degrees to true ones. This is great, but I wonder about the origin of this function. In my understanding, it is a modified version of a function from another package, confusionMatrix, written by Michael Clark (not an author of the paper). Several related functions appear to be copied verbatim into skater. All of this is clearly marked in the code, with Clark rightfully listed as author, and Clark's package does come with a permissive license (MIT), but I still find it a bit odd. Why not simply import it? That way, bug fixes and improvements in the original version would automatically propagate to skater, avoiding the confusion of multiple versions. If the problem is that confusionMatrix is not on CRAN, perhaps one could reach out to make this happen?
4. In the example illustrating `confusion_matrix()`, the authors construct a dataset by modifying a previous one, by "randomly flipping ~20% of the true degrees". The term "flip" sounds misplaced to me here, since the variable isn't dichotomous. Also, the procedure doesn't change 20%, only 0.2 * 4/5 = 16% on average, since the new values are generated from the complete set and stay unchanged with probability 1/5. Regardless of these minor issues, I must admit I found the example rather artificial. It would be much more interesting to see confusion_matrix() applied to a real dataset! That could also motivate some comments on how its output should be interpreted.

Finally, I cannot resist offering a couple of suggestions for the skater package itself:

1. The `read_fam()` for reading .fam files is very strict, insisting on space-separated columns and disallowing any format deviations. I note that e.g. PLINK, and several other R packages that read pedigree files, allow variations like tab-separated columns and missing phenotype column; perhaps this could be useful in the skater package too.
2. Separating parent-offspring pairs from full sibs is a crucial step in many relatedness studies, and often possible by a simple analysis of the output of e.g. KING. Does skater offer such differentiation? If not, it might make a nice addition to the package in the future.

Are the conclusions about the tool and its performance adequately supported by the findings presented in the article?

Partly

Is the rationale for developing the new software tool clearly explained?

Yes

Is the description of the software tool technically sound?

Yes

Are sufficient details of the code, methods and analysis (if applicable) provided to allow replication of the software development and its use by others?

Yes

Is sufficient information provided to allow interpretation of the expected output datasets and any results generated using the tool?

Yes

Reviewer Expertise:

Statistical genetics, pedigree analysis, R

I confirm that I have read this submission and believe that I have an appropriate level of expertise to confirm that it is of an acceptable scientific standard, however I have significant reservations, as outlined above.

# Peer review: Reviewer response for version 1

**DOI:** 10.5256/f1000research.79953.r119026

**Published:** 2022-01-19

**Authors:** Wei-Min Chen

**Affiliations:** 1 Center for Public Health Genomics, University of Virginia, Charlottesville, VA, USA

The authors developed an R package to evaluate the performance of different relatedness inference tools. Both the R package and the manuscript are user-friendly and easy to follow. This tool should be timely and valuable to geneticists (or data analysts) who have a need to compare different relatedness inference tools and choose a tool that performs the best for their own genetic datasets. The manuscript is very well written.

 One very minor comment is about the format requirement for the developed tool. Is the required format basically PLINK with expectations that some commonly used PLINK formats are still not allowed, or all PLINK format is acceptable? E.g.,

1. How about only one parent available, with Father ID 1234 and mother ID 0? This is allowed and considered as PLINK format. How does the proposed tool handle it, e.g., ask the user to reformat it?
2. How about both parents only with IDs available. E.g., Father ID is 1234 and mother ID is 5678, and none of them have appeared again in Individual IDs? 

Again, the comment above is quite minor and may only require some clarification. The overall quality of this manuscript is great.

Are the conclusions about the tool and its performance adequately supported by the findings presented in the article?

Yes

Is the rationale for developing the new software tool clearly explained?

Yes

Is the description of the software tool technically sound?

Yes

Are sufficient details of the code, methods and analysis (if applicable) provided to allow replication of the software development and its use by others?

Yes

Is sufficient information provided to allow interpretation of the expected output datasets and any results generated using the tool?

Yes

Reviewer Expertise:

My area of research is statistical genetics, specifically developing methods and tools for relatedness inference.

I confirm that I have read this submission and believe that I have an appropriate level of expertise to confirm that it is of an acceptable scientific standard.
